# Probiotics Upregulate Trefoil Factors and Downregulate Pepsinogen in the Mouse Stomach

**DOI:** 10.3390/ijms20163901

**Published:** 2019-08-10

**Authors:** Ghalia Khoder, Farah Al-Yassir, Asma Al Menhali, Prashanth Saseedharan, Subi Sugathan, Catherine Tomasetto, Sherif M. Karam

**Affiliations:** 1 Department of Pharmaceutics and Pharmaceuticals Technology, College of Pharmacy, Sharjah Institute for Medical Research, University of Sharjah, Sharjah 27272, UAE; 2 Department of Anatomy, College of Medicine and Health Sciences, United Arab Emirates University, AlAin 17666, UAE; 3 Department of Biological Sciences, Faculty of Science, Beirut Arab University, Debbieh Campus PO Box 11-50-20 Riad El Solh, Beirut 11072809, Lebanon; 4 Department of Biology, College of Science, United Arab Emirates University, Al-Ain 15551, UAE; 5 Institut de Génétique et de Biologie Moléculaire et Cellulaire (IGBMC), Institut National de la Santé et de la Recherche Médicale (INSERM), U1258, Centre National de la Recherche Scientifique (CNRS), UMR7104, Université de Strasbourg, F-67404 Illkirch, France

**Keywords:** probiotics, VSL#3, trefoil factor, mucin, ghrelin, pepsinogen, gastric epithelium

## Abstract

Probiotics are used in the management of some gastrointestinal diseases. However, little is known about their effects on normal gastric epithelial biology. The aim of this study was to explore how the probiotic mixture VSL#3 affects gastric cell lineages in mice with a special focus on protective and aggressive factors. Weight-matching littermate male mice (*n* = 14) were divided into treated and control pairs. The treated mice received VSL#3 (5 mg/day/mouse) by gastric gavage for 10 days. Control mice received only the vehicle. Food consumption and bodyweight were monitored. All mice were injected intraperitoneally with bromodeoxyuridine (120 mg/Kg bodyweight) two hours before sacrificed to label S-phase cells. Stomach tissues were processed for lectin- and immunohistochemical examination. ImageJ software was used to quantify immunolabeled gastric epithelial cells. Real-time quantitative polymerase chain reaction was used to provide relative changes in expression of gastric cell lineages specific genes. Results revealed that treated mice acquired (i) increased production of mucus, trefoil factor (TFF) 1 and TFF2, (ii) decreased production of pepsinogen, and (iii) increased ghrelin-secreting cells. No significant changes were observed in bodyweight, food consumption, cell proliferation, or parietal cells. Therefore, VSL#3 administration amplifies specific cell types specialized in the protection of the gastric epithelium.

## 1. Introduction

Probiotics are microorganisms that exert beneficial effects on their hosts when ingested in sufficient amounts [1]. The most prevalent probiotics used are lactic acid bacteria and bifidobacteria, although other bacteria and yeasts are also used [2]. Ingestion of probiotics helps in maintaining the balance of intestinal microflora and in reducing the side effects of antibiotics [3]. Some studies have shown that probiotics are safe and their use has no side effects [4]. Therefore, probiotics are now incorporated into an expanding array of food products, nutritional supplements, and pharmaceutical compounds [5].

In the last decades and with the great discovery of the next generation sequencing technique, several studies have emerged to explore the beneficial effects of probiotics on human diseases [5,6], including cancer [7,8,9]. In the gastrointestinal tract, it has been shown that probiotics have potential roles in the management of inflammatory, ulcerative, and neoplastic diseases [10,11,12]. When administered, probiotics compete with pathogenic bacteria for nutrients [13], and contribute to the stability of the intestinal epithelial barrier including tight junctions [14]. 

Probiotics are provided as mono-strain [15,16] or multi-strain, such as the product called VSL#3 or simply VSL, which is a mixture of highly concentrated lyophilized living bacteria [17]. Studies have shown that multi-strain probiotics are more effective than mono-strains and have more successful chances of colonization [18] due to the ability to overcome adverse physiological conditions of the gut such as acidic pH, digestive enzymes, bile acid, and mechanical stress.

VSL has been extensively investigated in the last few years [9,19,20,21]. Currently, VSL is recommended for the prevention and treatment of inflammatory bowel diseases [22], such as pouchitis [23], Crohn’s disease [24], and ulcerative colitis [21,25,26]. A recent study has also revealed the preventive effect of VSL in ulcerative colitis-associated carcinogenesis in mice [9]. With all these advances in the basic knowledge and clinical applications of probiotics in gastrointestinal disorders, little is known about their mechanism of action in the lower gastrointestinal tract [27]. Modulation of host-microbe interactions, pathogen exclusion, stimulation of goblet cells to produce mucus, enhancing intestinal epithelial barrier formation, production of antibacterial factors, and activation of host adaptive immune response have been proposed [28,29]. 

Several studies, about the impact of probiotic bacteria to health, notably host mucosal defense responses, have shown potentials in reinforcing epithelial integrity, modulation of barrier function, and upregulation of adaptive defense responses such as secretion of mucins and intestinal trefoil factor (TFF3) [30]. It was also reported that under inflammatory conditions, probiotic administration normalized the abnormal stimulation of mucus secretion [31,32]. Several studies have demonstrated the potent effect of probiotics in mucin gene expression in colonic and intestinal epithelial cell lines [33,34] and in animal models [35]. In addition, an interesting study on the effect of VSL administration on acetic acid-induced gastric ulcer in rats reported that the main impact for VSL was on the upregulation of vascular endothelial growth factor [19]. However, the effects of VSL on the gastric epithelial cell lineages of the healthy stomach are not yet explored. 

The stomach is lined by an epithelial cell layer organized to form numerous pits lined by mucous cells, and continuous with tubular glands populated with multiple cell lineages producing mucus, acid, pepsinogen, and several hormones. These cell lineages originate from proliferating epithelial stem cells [36]. This study aims to investigate whether VSL administration into mice affects gastric epithelial cell biology with specific reference to the defensive and aggressive factors of the gastric epithelium. 

## 2. Results

All seven pairs of young male control mice and their littermates exposed to VSL were physically active during the 10 days of gastric gavages. However, measurements of various body and gastric tissue parameters revealed some important observations. 

### 2.1. VSL Administration Has No Significant Effect on Mouse Bodyweight and Food Intake

**Figure 1:**
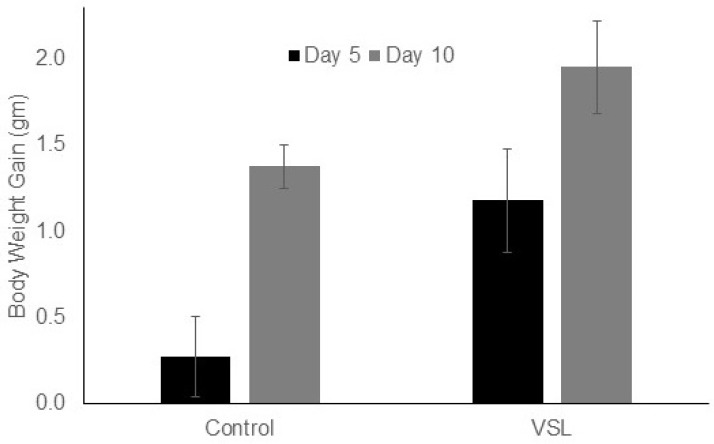
Effect of VSL on mice bodyweight gain in grams after five and 10 days of administration. VSL treatment shows an increasing trend in bodyweight gain after treatment but was not statistically significant when compared to control. Data from seven control and seven VSL-mice are presented as mean ± SD.

The bodyweight of VSL-treated mice showed slightly higher values as compared to control mice. After five days of VSL administration, the bodyweight was 28.7 ± 0.32 gm on the average as compared to 26.6 ± 0.31 gm in control mice. By day 10, the bodyweight averaged 29.3 ± 0.25 gm in VSL-mice and 26.9 ± 0.29 gm in control mice. This increase in weight gain of VSL-mice was not statistically significant. Estimation of bodyweight gain after 10 days in each pair of control and VSL-treated mice showed that the percentages of increase in weight gain were 5.2% and 8.3%, respectively (Figure 1). Estimating the weight of food consumed by mice after five and 10 days revealed slight increase (1.2-fold) in the cumulative food intake that was not statistically significant.

### 2.2. VSL Enhances Mucous Production in the Mouse Gastric Mucosa

Tissue sections of the gastric mucosae of control and VSL-treated mice stained with conventional hematoxylin and eosin showed no apparent cellular differences. Both groups of tissues had long oxyntic glands with large scattered eosinophilic parietal cells. The VSL-treated tissues had a tendency to have some increase in mucosal thickness and glandular length. Measurements of the glandular mucosal thickness in a comparable region (close to the fundus) of tissue sections of control and VSL-exposed stomachs showed values that varied from 312 to 423 µm in control and 401 to 412 µm in VSL-treated tissues. This difference was not statistically significant (*p* > 0.05). Similar insignificant differences were also found for two other comparable regions of the corpus mucosa. While conventional microscopy did not reveal significant differences, systematic molecular examination using a variety of microscopy approaches demonstrated several differences related to the epithelial cell lineages.

**Figure 2:**
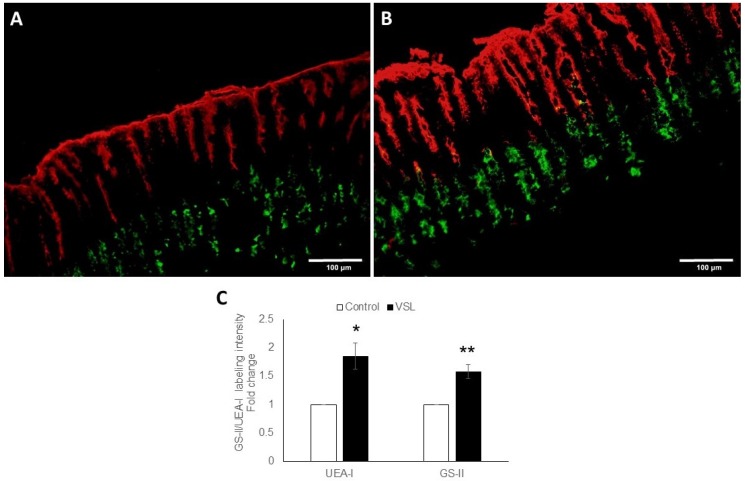
Double lectin histochemistry of the gastric mucosae of control and VSL-treated tissues. Stomach tissue sections of control (**A**) and VSL-treated (**B**) mice were incubated with UEA-I (red) and GS-II (green). Images obtained with 20× objective lense, scale bar: 100 µm. Fold changes in the quantification of the UEA-I and GS-II labeling intensity of control (*n* = 7) and VSL-treated (*n* = 7) tissues (**C**) are presented as mean ± SE. The *asterisks* indicate significant differences from the control group. * *p* <0.05, ** *p* < 0.01.

To examine the amount of mucus in both pit and neck cell lineages, double lectin binding was employed, and revealed the presence of more mucus in the VSL-treated tissues than in control (Figure 2A,B). Measurements of UEA-lectin labeling in each pair of control and VSL treated mice showed an increased labeling in VSL treated tissues by 1.84 ± 0.23 folds (*p* < 0.05) (Figure 2C). Similarly, there was intensified GSII-lectin labeling of neck cells in VSL-treated mice when compared with control tissues and the fold increase was 1.58 ± 0.12 (*p* < 0.01; Figure 2C). To eliminate possible processing variations, tissue sections of control and VSL-mice were processed together for embedded in the same paraffin block, simultaneously exposed to the same immunolabeleing solutions, and photgraphed with the same microscopy settings.

### 2.3. VSL Enhances TFF1 and TFF2 Production by Gastric Mucous Cells

**Figure 3:**
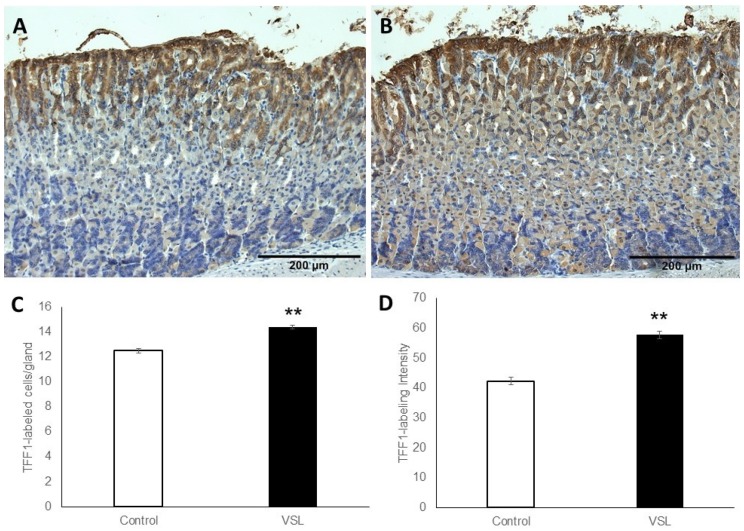
Immunohistochemical analysis of TFF1 in gastric mucosal tissue sections obtained from control and VSL-treated mice. Note that TFF1-labeled epithelial cells (brown cytoplasm) of VSL-treated tissues in (**B**) appear more expanded at the pit region of gastric gland when compared with control tissues in (**A**). Magnification, 20×, scale bar: 200 µm. Analysis of TFF1-labeled cell counts per gland in the gastric corpus of control and VSL-treated mice (**C**), and TFF1-labeling intensity per field in the oxyntic glands of control and VSL treated mice (**D**). Data from four control and four VSL-mice are presented as mean ± SE. The *asterisk* indicates significant differences from the control group. ** *p* < 0.01.

**Figure 4:**
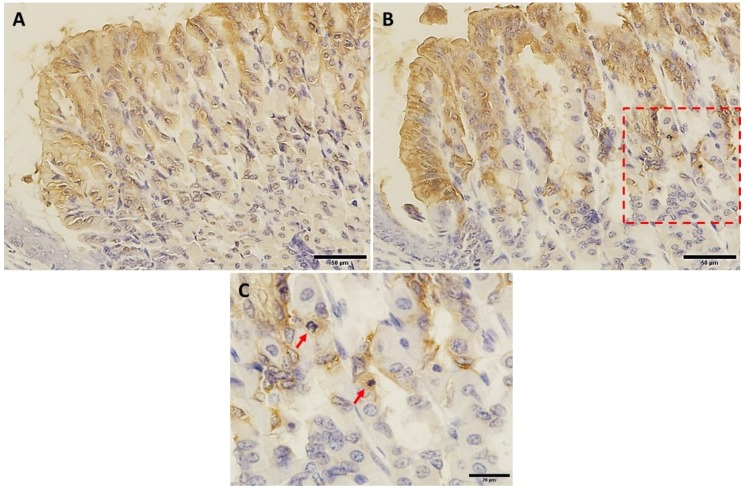
TFF1-labeled epithelial cells in corresponding regions of control (**A**) and VSL-treated (**B**) gastric mucosae at high magnifications. Both A and B are taken from the same region at the junction with the stratified epithelium of the fundus (lower left corner). Note the higher intensity of the brown color of TFF1-labeling in VSL-treated tissue (**B**) compared to control (**A**). Magnification bar = 50 µm. Panel (**C**) shows a high magnification of the area within the red square of the VSL-tissue. Note the TFF1-labeled mitotic progenitor cells at the arrows. Magnification bar = 20 µm.

To test whether increased mucus was associated with a change in the production of trefoil factors, tissues were probed with antibodies specific for TFF1 and TFF2. The labeling for TFF1 occurred in the pit cells lining the luminal surface and along the pit regions (Figure 3A,B). Measurements revealed increased labeling for TFF1 in VSL-tissues when compared to the control (Figure 3C). In addition, counts of nuclei of TFF1-labeled cells indicated the presence of 12.4 ± 0.19 and 14.4 ± 0.14 cells per gland in the control and VSL-treated mice, respectively (*p* < 0.01, Figure 3C). Quantification of the percentage of pixels in labeled areas in images obtained with 20× magnification also revealed a significant increase in the intensity of TFF1-labeling of VSL-treated tissues (Figure 3D). To confirm the increase of the TFF1 level, images obtained from the same region of the corpus of both control and VSL tissues were compared at higher magnification (Figure 4A,B). Interestingly, the increased level of TFF1-labeling was not only demonstrated in surface and pit cells of VSL-treated tissue, but also dividing progenitor cells of the isthmus region showed some cytoplasmic staining for TFF1 (Figure 4C). 

**Figure 5:**
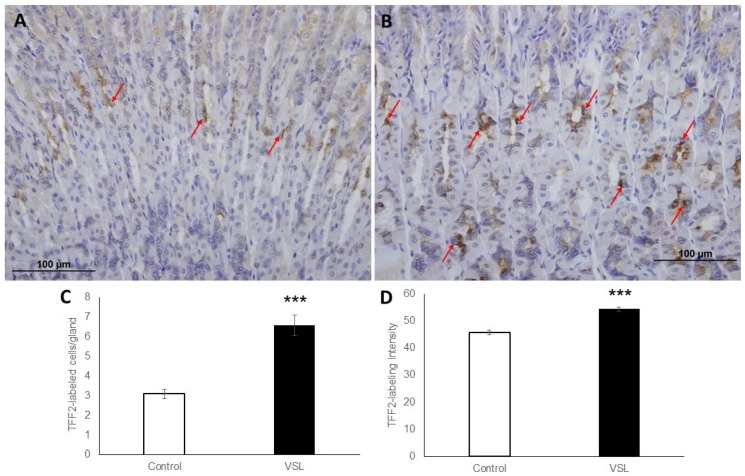
Immunohistochemical analysis of TFF2-labeled epithelial cells in tissue sections obtained from the gastric mucosa of control (**A**) and VSL-treated (**B**) mice. Magnification bar = 100 µm. Analysis of TFF2-labeled cell counts per gland in the gastric corpus of control and VSL-treated mice (**C**), and TFF2-labeling intensity per field in the oxyntic glands of control and VSL treated mice(**D**). Data from four control and four VSL-mice are presented as mean ± SE. The asterisks indicate significant differences from the control group. *** *p* < 0.001.

**Figure 6:**
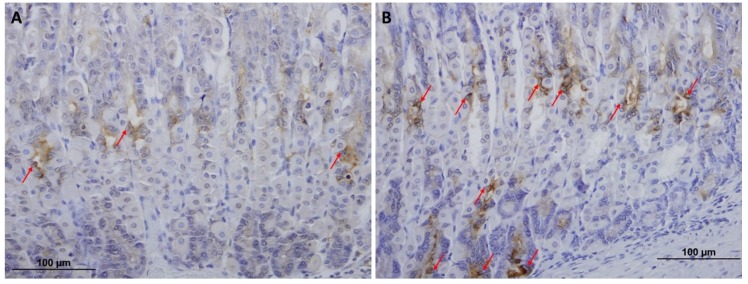
TFF2-labeled epithelial cells (arrows) in tissue sections obtained from the middle and basal portions of the gastric mucosae of control (**A**) and VSL-treated (**B**) mice. Note the extension of TFF2 labeling to the bottom of the gastric glands where chief cells (lower three arrows) are located. Magnification bar = 100 µm.

To determine whether the level of TFF2 produced by mucous neck cells was also affected by VSL administration, mucosal sections were probed with anti-TFF2 antibodies. Sections of both control (Figure 5A) and VSL-treated tissues (Figure 5B) demonstrated the presence of TFF2 in the neck region. To provide quantitative data for the number of TFF2-labeled neck cells, three different images from four different control and VSL-mice were examined. For each animal, counts in longitudinally cut glands were averaged and expressed as cells per gland. Data showed a significant difference (*p* < 0.001) in the number of TFF2-labeled cells in VSL-treated mice (6.5 ± 0.5) when compared to controls (3.09 ± 0.2; Figure 5C). Quantification of the percentage of pixels in labeled areas in images obtained with 20× magnification also revealed a significant increase in the intensity of TFF2 labeling of VSL-treated tissues (Figure 5D). In addition, the labeling for TFF2 extended deep in the gastric glands and in some glands reached to the bottom where chief cells are located (Figure 6A,B).

### 2.4. VSL Inhibits Pepsinogen Production by Chief Cells

**Figure 7:**
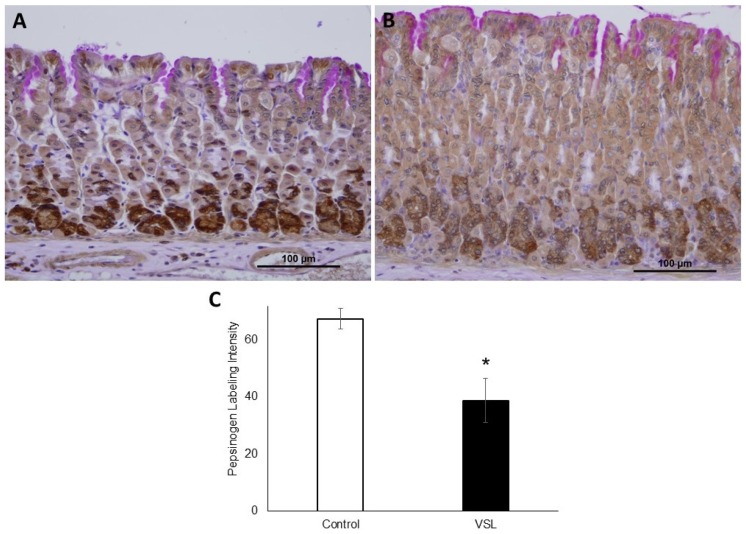
Immunohistochemical analysis of pepsinogen immunolabeling in tissue sections obtained from the gastric mucosae of control (**A**) and VSL-treated (**B**) mice. Magnification bar = 100 µm. Analysis of pepsinogen-labeling intensity per field in the oxyntic glands of control and VSL treated mice (**C**). Data from three control and three VSL-mice are presented as mean ± SE. The asterisk indicates significant differences from the control group. * *p* < 0.05.

**Figure 8:**
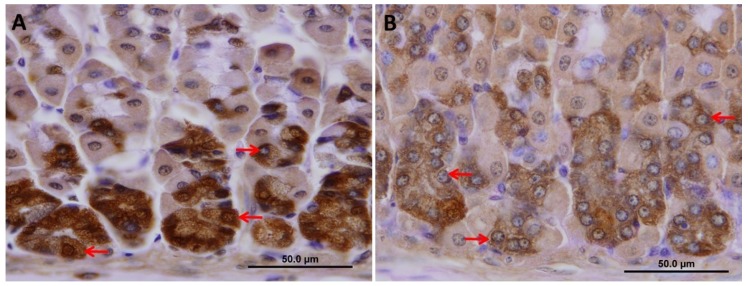
A high magnification micrograph of pepsinogen immunolabeling in tissue sections obtained from the gastric mucosae of control (**A**) and VSL-treated (**B**) mice. Note the reduced pepsinogen labeling in chief cells of VSL mice at the bottom of gastric glands. These cells tend to appear smaller than those of control with a less amount of secretory granules (arrows in A vs. B). Magnification bar = 50 µm.

To test whether increased TFF2 production was associated with alteration in chief cells, tissues were probed with antibodies specific for pepsinogen (Figure 7A,B). It was interesting to find out that the intensity of pepsinogen labeling was significantly reduced after the 10-day treatment with VSL (Figure 7C). In addition, a close-up examination of tissue sections showed that the reduced pepsinogen labeling in chief cells of VSL mice at the bottom of gastric glands was associated with a decreased cellular size (Figure 8A,B). 

### 2.5. VSL Treatment neither Affect Cell Proliferation nor H^+^,K^+^-ATPase Immunolabeling

**Figure 9:**
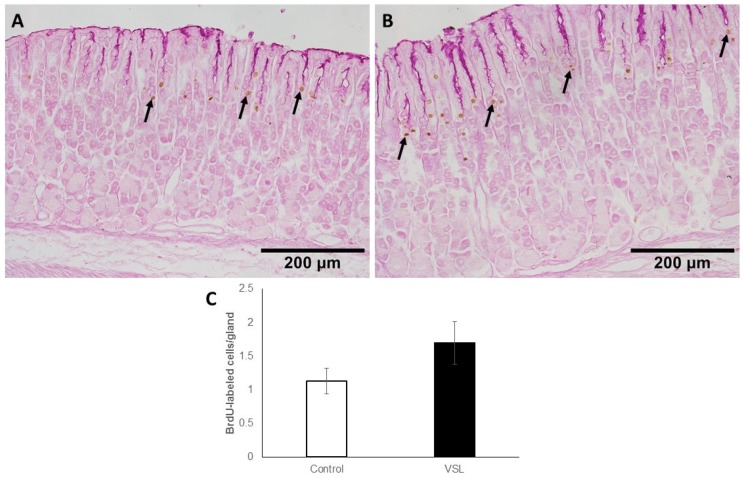
Immunohistochemical analysis of S-phase cells using anti-5′-bromo-2′-deoxyuridine (BrdU) antibody in the gastric mucosa of control (**A**) and VSL-treated (**B**) mice. Note that BrdU-labelled cells (brown nuclei) of VSL-treated tissues tend to appear more expanded when compared with control tissues and uniformly distributed in most of the glands. Magnification bar = 200 µm. Analysis of BrdU-labeled cell counts per gland in the mucosa of seven control and seven VSL-treated mice are presented as mean ± SE (**C**). No significant difference was found between VSL and control tissues.

The gastric mucosa of control mice showed a few BrdU-labeled cells at the pit-gland junctions close to the luminal surface (Figure 9A). However, in the VSL-treated mice, the area of pit-gland junction tended to show more dividing cells (Figure 9B). Quantification of BrdU labeled cells in three different equivalent images of three control and three VSL mice revealed the presence of 1.13± 0.18 and 1.7 ± 0.31 cells per gland, respectively (Figure 9C). The difference was not statistically significant (*p* > 0.05). 

**Figure 10:**
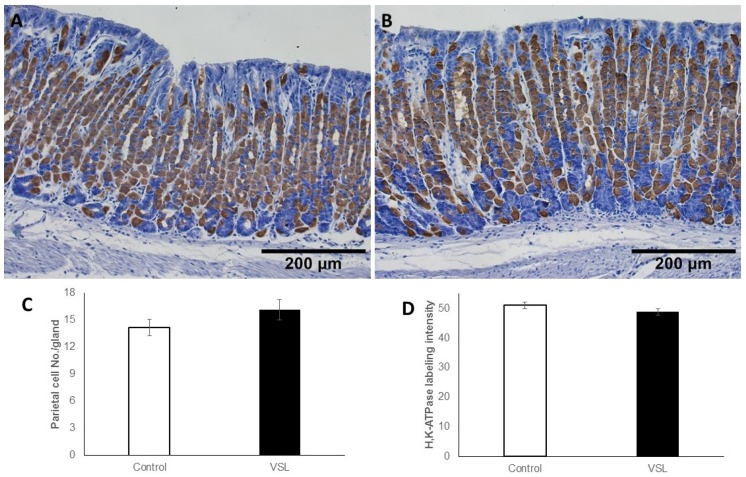
Immunohistochemical labeling of parietal cells in the gastric mucosa of control (**A**) and VSL-treated (**B**) mice with antibodies specific for H^+^,K^+^-ATPase β-subunit. Labeled parietal cells are distributed throughout the gastric glands. Magnification bar = 200 µm. Quantification of H^+^,K^+^-ATPase-labeled cells per gland (**C**) and the labeling intensity per field (**D**) in seven control and seven VSL-mice are presented as mean ± SE. No significant differences were observed.

As for parietal cells, incubation with antibodies specific for the β-subunit of H^+^,K^+^-ATPase demonstrated the scattered distribution of parietal cells in both control (Figure 10A) and VSL-treated tissues (Figure 10B). Counts showed that parietal cells averaged 14.21 ± 0.91 cells per gland in control and 16.17 ± 1.13 cells in the VSL-treated tissues (Figure 10C). When the intensity of the H^+^,K^+^-ATPase labeling was compared in both groups of tissues, no apparent difference was noted. Quantification of the percentage of pixels in labeled areas in images obtained with 20× objective revealed no significant difference (*p* > 0.05).

### 2.6. VSL Treated Tissues Showed an Increase in Ghrelin-Secreting Cells

**Figure 11:**
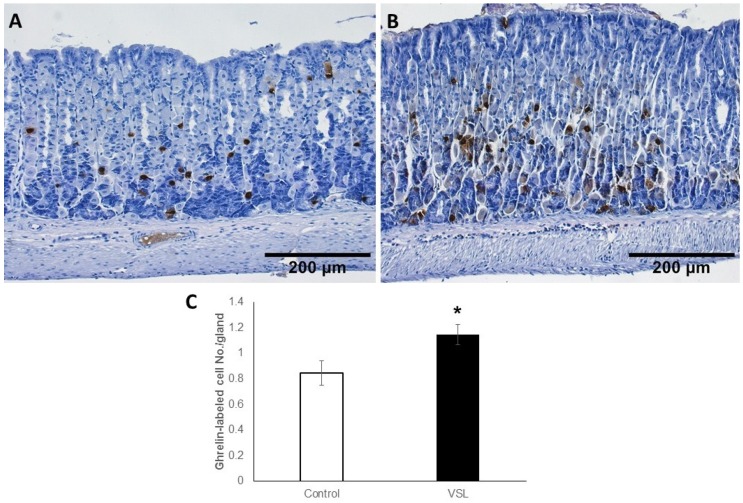
Immunohistochemical analysis of ghrelin-secreting cells in the gastric mucosa of control (**A**) and VSL-treated (**B**) mice. Note the labeled cells scattered along the gastric glands. Magnification bar = 200 µm. Counts of ghrelin-labeled cells per gland in six control and six VSL-mice are presented as mean ± SE (**C**). The *asterisk* indicates significant increase in VSL-tissues as compared to control. * *p* < 0.05.

For enteroendocrine cells, ghrelin antibodies were used since they label different subtypes of enteroendocrine cells in the gastric mucosa. Immunoprobed tissue sections of control (Figure 11A) and VSL-treated (Figure 11B) mice, revealed that ghrelin secreting cells were scattered along the gastric glands. Surprisingly, tissue sections obtained from the VSL-treated mice showed more ghrelin-labeled cells than in control. Counts revealed the presence of 0.8 ± 0.09 and 1.14 ± 0.07 cells per gland on the average in the gastric mucosa of control and VSL-treated mice, respectively (Figure 11C). This difference was statistically significant (*p* < 0.05).

### 2.7. Effects of VSL Treatment on the Expression of Various Gastric Cell Lineage-Specific Genes

**Figure 12:**
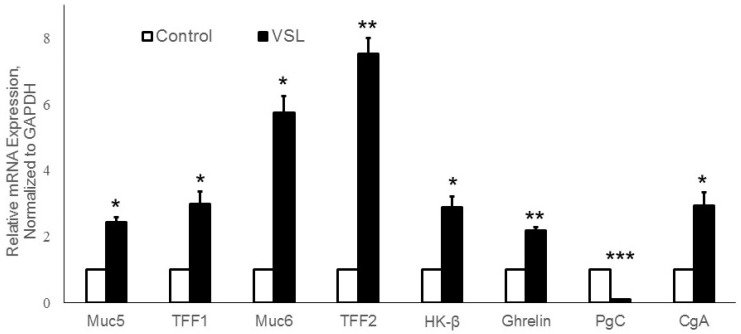
Quantitative RT-PCR analysis of mRNA expression of VSL–treated tissues versus control tissues. Expression of TFF1, TFF2, Muc5ac, Muc6, H^+^,K^+^-ATPase β subunit, ghrelin, CgA and PgC in VSL and control tissues were compared. Data from three different pairs of control and VSL-treated mice were presented as mean ± SE and normalized to GAPDH. The asterisks indicate significant differences from the control group. * *p* < 0.05; ** *p* < 0.01; *** *p* < 0.001.

To substantiate these findings using another method, gene expression levels of different gastric proteins/peptides including Muc5AC, Muc6, TFF1, TFF2, PgC, H^+^,K^+^-ATPase β-subunit, ghrelin, and CgA were measured by real-time quantitative polymerase chain reaction (RT-qPCR). The primers used are listed in Table 1. Analysis of the expression of Muc5ac gene-specific for mucus-secreting pit cells in VSL-treated mice showed a 2.5-fold increase when compared to control (*p* < 0.05, Figure 12). Treatment with VSL also showed 5.5-fold induction of Muc6 gene expression (*p* < 0.05, Figure 12). TFF1 and TFF2 expression analysis revealed a 3.5- and 7.5-fold increase in VSL-treated tissues (*p* < 0.05, *p* < 0.01, respectively, Figure 12).

To further assess the effect of VSL on other gastric cell lineages, a significant increase (2.9-fold) in H^+^,K^+^-ATPase β-subunit mRNA expression was found when compared to control (*p* < 0.05). This finding was not consistent with immunohistochemical analysis. However, consistent with immunohistochemistry, analysis of PgC expression of chief cells in VSL mice showed a significant reduction in mRNA abundance compared to control (*p* < 0.001, Figure 12). 

To test whether VSL administration would influence its effect on ghrelin expression, we measured the ghrelin gene expression in control as well as VSL groups. VSL group demonstrated ~2.2-fold enrichment in ghrelin mRNA expression. A 10-day period of VSL administration exhibited an abundant expression of ghrelin mRNA when compared to control, this upregulation was statistically significant (*p* < 0.01, Figure 12). Moreover, to confirm that VSL has an effect on gastric enteroendocrine cells, chromogranin A (*CgA*), a general marker for differentiated endocrine cells, was also tested. This analysis revealed approximately a 3.5-fold change in CgA gene expression on average in all VSL-treated mice when compared to controls. This upregulation in CgA gene expression in VSL-treated mice was significant (*p* < 0.05, Figure 12). Taken together, these results point to a clear effect for VSL administration on the gastric epithelium. VSL enhances gastric epithelial protection through upregulation of TFF1, TFF2, Muc5ac, Muc6, and ghrelin and downregulation of PgC. These findings will highlight the preventive and therapeutic values of probiotics in gastric diseases.

## 3. Discussion

Recently, much attention has been paid to the impact of probiotics in the gastrointestinal tract. However, the mechanisms through which probiotics exert their effect are still unclear. Several clinical studies have shown that probiotics modulate gut microflora [37,38] and protect the gastric mucosa through the regulation of cellular proliferation and gastric mucin production [33,35,39,40,41,42]. In our current study, the multi-strain probiotic mixture VSL was found to mainly upregulate expression and production of TFF1, TFF2, and mucins in the mouse stomach. Further investigations have shown that VSL administration also enhances the expression of ghrelin and chromogranin and production of enteroendocrine ghrelin-secreting cells. Interestingly, upregulation of TFF2 was associated with their production in some chief cells located at the bottom of the gastric glands. These chief cells not only expressed a lower level of pepsinogen gene and smaller amount of pepsinogen protein, but they appeared smaller in size as compared to those of control mice.

It should be stated that conventional histological examination of hematoxylin and eosin-stained stomach tissue samples from VSL-treated mice has not revealed morphological changes when compared to control mice. It was also reported previously that administration of *Lactobacillus acidophilus* and *Bifidobacterium* spp. had no effect on the morphology of the stomach and intestine of piglets [43]. However, molecular analysis using detailed immuno- and lectin-histochemical methods, as well as RT-qPCR, has revealed several changes. 

The mucus gel layer is a structural component of the gut that lubricates and protects the gastrointestinal tract against various harmful agents. In the present study, compelling evidence shows that VSL enhances mucus production. Both lectin staining and RT-qPCR for the expression of mucin genes (Muc5ac and Muc6) has supported this observation. The increased gastric mucous secretion is not surprising and coincides with other studies which demonstrated an increase in the thickness of the mucus gel layer of the gastric mucosa when treated with exopolysaccharide producing *Streptococcus thermophilus* CRL 1190 [44] or *Bifidobacterium bifidum* BF-1 [45] to alleviate the chronic gastritis in mice or acute gastric injury in rats. Similarly, it was demonstrated that treatment of ethanol-induced gastric mucosal lesions with *Lactobacillus rhamnosus* GG indirectly stimulated the mucous secretion and transmucosal resistance through the up-regulation of prostaglandin [40]. One study aimed to investigate the effect of the supernatant of milk fermented by *Lactobacillus helveticus* R239 on gut physiology and demonstrated an increase in the number of goblet cells, suggesting an increase in mucus secretion [46]. Moreover, several studies have demonstrated the potent effect of probiotics in mucin gene expression in colonic and intestinal epithelial cell lines [33,34] and in animal models [35]. On the other hand, contradictory studies were observed when VSL probiotic mixture was used in acetic acid induced gastric ulcer in rats [19] or in dextran-sodium sulfate-induced chronic colitis in mice [47]. These studies showed that VSL has either a modest increase in the expression of Muc5ac in a rat model of the acetic acid-induced gastric ulcer [19] or no potential role for mucin in dextran-sodium sulfate-induced chronic colitis in mice [47]. These findings would rather suggest the beneficial usage of VSL as a preventive agent more than a therapeutic agent.

TFFs are typical constituents of mucus-secreting epithelia. The increase in mucous secretion found in the present study coincided with an increase in the number of cells expressing TFF1 and TFF2. This result is not unexpected since TFFs co-localized and co-secreted with mucins [48,49]. Individual TFFs have also been noted to interact with specific mucins preferentially, and such interaction is important to strengthen the mucous barrier [50]. Accordingly, TFF peptides are considered as constituents of the mucus barrier, where they display lectin-like behavior [51]. TFF3 interacts with specific carbohydrate moieties and stabilizes the gastric mucous barrier [48,52,53]. TFF2 was found to protect the mucosa from insults by stabilizing the mucous layer and inhibiting acid secretion [48,54].

In normal conditions, TFFs are expressed in the gastrointestinal tract in a tissue-specific manner. In humans, TFF1 and TFF2 are expressed in the stomach and duodenum [55,56]. TFFs are considered to play critical roles in maintaining mucosal integrity and defense [57,58], promoting cell migration [59,60] and enhancing cell proliferation and differentiation [61,62]. This raises the proposal that TFFs are good therapeutic candidates for the treatment of several gastrointestinal diseases. While probiotics are considered as a promising therapy against gastrointestinal inflammation, only a few studies have addressed how probiotics associate with TFFs in the host defense. One of the most interesting findings in our study was the increase in the expression of TFF1 and TFF2 in the stomach of VSL-treated mice. Several studies have pointed to TFF peptides as crucial players in mucosal protection, repair, reconstitution, and differentiation [49,51,63]. In the present study, we demonstrated a proportional upregulation in TFF1 and TFF2 of all VSL-treated mice. These findings are coherent with other studies which demonstrated that oral administration of genetically recombinant TFF-secreting *Lactococcus lactis* exerted both prophylactic and therapeutic effects in the mouse model of acute colitis [64]. Another study has also demonstrated that supplementation of *Lactobacillus rhamnosus* GG supernatant increased the TFF3 in alcohol damaged Caco-2 cell and also in a mouse model of alcohol-induced liver disease [65,66]. Conversely, another study indicated that LGG supplementation in the diet did not show significant regulatory effects in the gene expression of TFFs in mice with induced colitis [67]. Additionally, in a rat model of neonatal necrotizing enterocolitis, the numbers of TFF3 positive cells were reduced to normal level after feeding with live *Bifidobacterium bifidum* [31]. Other studies have also demonstrated that supplementation of *Enterococcus faecium* has no effect on intestinal TFF3 expression in animal models [68,69]. Further studies are needed for better understanding of the role of probiotics and TFFs and how their interactions might affect the host defense.

In the present study, the immunolabeling of TFF2 was not only detected in the middle of the gastric glands where mucous neck cells are located but were frequently detected at the bottom of the glands where pepsinogen-secreting chief (or zymogenic) cells dominate. This interesting finding correlated with the downregulation of pepsinogen gene expression and immunolabeling in the gastric mucosa of VSL mice. Since it has been established that neck and zymogenic cells belong to the same cell lineage [36], this finding could be explained in different ways. As a consequence of VSL administration, either (i) neck cells did not continue to differentiate into fully mature zymogenic cells and have remained as pre-zymogenic cells producing mucin, TFF2 and pepsinogen, or (ii) zymogenic cells started to dedifferentiate into pre-zymogenic cells which acquired some mucin, TFF2, but lost some of its machinery to produce pepsinogen. In either case, the reduction in pepsinogen and increase of mucus and TFF2 indicates a profound gastroprotective effect for VSL on the gastric mucosa. The decrease in pepsinogen in VSL-treated mice is in agreement with another study that demonstrated a decrease in pepsinogen I/II ratio in humans after 12 weeks of treatment with *Bifidobacterium bifidum* fermented milk [70]. 

By using BrdU labeling method, this study demonstrates that VSL has no effect on gastric epithelial cell proliferation. To eliminate the possible animal variations, VSL-treated and control mice were not only littermates but also sex- and weight-matched. In control and VSL-tissues, a few BrdU-labeled cells were localized in the isthmus regions of the gastric glands. These findings are in agreement with a previous study of Lam et al., 2007 who demonstrated that treatment with *Lactobacillus rhamnosus* GG reduced cellular apoptosis in the rat gastric mucosa with ethanol induced lesions but did not report any effect on cellular proliferation [40]. Conversely, VSL-treated rats showed a significant increase in the proliferation index in the colon compared to control [27]. In addition, VSL showed antiproliferative effect against cancer cell lines (Jurkat, HT1080, and Caco-2 tumor cell lines) [71]. A recent study using the same VSL probiotic mixture has demonstrated epithelial regenerative effects in the small intestine and colon of mice [72]. Therefore, results on the effects of VSL on cell proliferation from previous studies are contradictory. This could be explained by the fact that the VSL probiotic mixture used in the previous studies might not be the same. Currently, two commercial VSL probiotic mixtures exist in the market. The original VSL form is produced in USA, while the newfound VSL form is produced in Italy and commercialized by European countries (UK and Holland). It was already published that the outcomes of both forms are not similar [71]. The VSL form used in the current study was the original form.

As for acid secreting cells, the current finding indicates that VSL induces about a 3-fold increase in the expression of the regulatory H^+^,K^+^ATPase β subunit gene. This may correlate with the previous findings on the possible use of probiotics in cancer. It is known that gastric cancer is associated with loss of parietal cells, so upregulation of the HK-β subunit gene could be seen as a preventive effect against cancer. However, the immunohistochemical analysis did not show any significant difference in the counts and labeling intensity of parietal cells. Further investigations are needed to clarify this intriguing result.

It is known that gastrointestinal microbiota, probiotics, and heat-killed microbes can regulate intestinal immunity. However, their effect on the secretion of gastrointestinal hormones is not well explored. An important finding of this study was not only upregulation of ghrelin gene expression in VSL-treated mice, but also the increase in the number of ghrelin-secreting cells. Coherent results were obtained by both immunohistochemistry and RT-qPCR. Only a few studies have investigated the effect of probiotics on ghrelin expression of the gastric mucosa [73,74,75]. Our results were consistent with the recent study that demonstrated that supplementation of Lactogen microgranules probiotic formula containing *Lactobacillus rhamnosus* increases significantly the ghrelin gene expression in fish larvae [72]. Interestingly, it was also demonstrated recently that oral administration of heat killed *Lactobacillus brevis* SBC8803 increased acyl ghrelin serum level in rats. In the same study, in vitro analysis of mouse primary stomach cells treated with the same probiotic also demonstrated induction of ghrelin secretion [74]. Not only ghrelin-secreting cells, but other enteroendocrine cells play essential roles in gut chemo-sensing to orchestrate appropriate functional responses of the host’s physiology and translate signals coming from the gut microbiota through their hormonal secretions [76,77]. In the present study, Quantitative PCR revealed that the VSL-fed mice increased the expression levels of not only ghrelin but also CgA. Recently, another study has also shown that probiotic strain *Escherichia coli Nissle* 1917 increased CgA mRNA expression of enteroendocrine cells in piglets [78]. Since ghrelin stimulates appetite, this explains the slight increase (even though insignificant) in the gain of bodyweight in VSL-treated mice. This finding requires further investigation perhaps by increasing the dose or the duration of VSL administration to find out whether a significant increase in bodyweight will occur.

Although the mechanism of action of probiotics in the mouse stomach tissue appears to be diverse, still there is a need for additional studies to fully understand the mechanistic strategy by which probiotics modulate various gastric cell lineages. This will hopefully provide greater opportunities for improving prevention and therapeutic strategies for gastric disorders. 

## 4. Materials and Methods

### 4.1. Animals

Animal Research Ethics Committee at UAE University approved the protocols used in this study (ERA_2016_5487). Fourteen male C57Bl/6 mice aged from three to five months and weighing 25 g on average were used in this study. The mice were used in two different experiments carried out independently. In the first experiment, mice were divided into two groups, three control and three VSL-treated. In the second experiment, each group included four mice. In each experiment, mice were paired, and each pair was a weight matched littermate. All mice were given *ad libitum* access to laboratory chow and water. Mice were kept under a 12 h light/dark cycle at room temperature (22–24 °C). 

### 4.2. Experimental Design

Each pair of mice included a treated and a control mouse. The treated mice (*n* = 7) received, by gastric gavage, the VSL#3 probiotic mixture (Sigma-Tau Pharmaceuticals, Inc., Gaithersburg, MD, USA). The VSL is composed of a mixture of highly concentrated lyophilized living bacteria (450 billion bacteria per sachet) of four species of *Lactobacilli*: *Lactobacillus acidophilus*, *Lactobacillus parcasei*, *Lactobacillus plantarum,* and *Lactobacillus delbrueckii* Subsp. *Bulgaricus*; three species of *Bifidobacteria*: *Bifidobacterium infantis*, *Bifidobacterium longum,* and *Bifidobactrium breve* and one species of *Streptococcus salivarius* Subsp *thermophilus sp*. The preparation and dose of VSL were according to previously published information [79]. VSL was suspended in water and given at a dose equivalent to 5 mg/day/mouse for 10 days. The control mice (*n* = 7) received only the vehicle. The bodyweights of all mice were measured on days 0, 5, and 10 of the experiment. The food intake during the 10-day experimental period was also estimated. To label dividing cells in the S-phase of the cell cycle, all mice received a single intraperitoneal injection of BrdU (120 mg/kg bodyweight) two hours before sacrifice. The stomachs were removed under anesthesia and processed for histological, immunohistochemical and RT-qPCR analyses.

### 4.3. Histological, Lectin Histochemistry, and Immunohistochemical Analysis

To examine the structure of the gastric mucosa, the posterior walls of the stomachs of the mice in each of the two groups were processed together for routine histological examination. The tissues were immediately immersed overnight in Bouin’s solution and then processed for paraffin embedding. To ensure equal conditions, tissues of each group of VSL-treated and their littermate control pairs were embedded together in the same paraffin block. Five-µm-thick tissue sections were stained with hematoxylin and eosin or periodic acid Schiff (Abcam, Cambridge, UK) and examined with the Olympus microscope connected to DP70 digital camera.

To label surface mucous cells and neck mucous cells, gastric tissues were deparaffinized, rehydrated, and incubated with blocking buffer (1% BSA, 0.5% Tween-20 in PBS) for 45 min at room temperature, and then incubated in fucose-specific *Ulex europaeus* agglutinin I (UEA-I) lectin (Vector Laboratories, United States) conjugated to rhodamine for 1 hr. Following PBS washes, tissues were incubated for 1 hr with N-Acetyl-D-glucosamine-specific *Griffonia simplicifolia* II (GS-II) lectin (Thermo-Fisher Scientific, Molecular probes by Life Technologies, Eugene, OR, USA) conjugated to fluorescein isothiocyanate (FITC). The tissue sections were washed in PBS and mounted with fluoro-shield mounting medium with 4’,6-diamidino-2-phenylindole (Abcam, Cambridge, UK). 

To localize TFF1, TFF2, chief cells, proliferating cells, parietal cells, and ghrelin-secreting cells, tissue sections were deparaffinized, rehydrated and washed in PBS. The endogenous peroxidase activity was blocked by incubation in 3% hydrogen peroxide for 35 min. All tissue sections on the slide (representing three or four pairs of control and VSL stomachs) were circled with a hydrophobic film using a PAP pen (Dako, Glostrup, Denmark). To block non-specific binding sites, the sections were incubated in 1% BSA containing 0.5% Tween-20 and PBS for 45 min. The sections were then incubated for 1 hr using mouse monoclonal anti-BrdU antibody (Medical and Biological Laboratories Co., Nagoya, Japan) or for overnight at 4 °C with mouse monoclonal anti-pepsinogen C (Abcam, Cambridge, UK), or anti-H^+^,K^+^-ATPase β-subunit (Medical and Biological Laboratories Co. Woburn, MA, USA), or anti-ghrelin [raised in the IGBMC laboratories, Strasbourg, France] antibodies, or rabbit polyclonal anti-TFF1 and anti-TFF2 antibodies [57]. Following PBS washes, the tissue sections were incubated with biotinylated donkey anti-mouse immunoglobulin G for 1 hr. Tissues were washed in PBS and then incubated in peroxidase-conjugated extravidin (Sigma, St. Louis, MO, USA). The antigen-antibody binding sites were revealed by using 3,3’-diaminobenzidine tetrahydrochloride (Sigma, St. Louis, MO, USA).

### 4.4. Immunohistochemical Quantification

To estimate the number of cells immunolabeled with antibodies specific for TFF1, TFF2, BrdU, H^+^,K^+^-ATPase, and ghrelin, the Fiji ImageJ software was used. At least three JPEG images obtained at 20× objective lens representing different fields with the best longitudinal orientation of the gastric gland were examined in each control and VSL-treated tissues. In each image, the labeled cells were tracked using the cell counter of the software and the total number of glands was manually counted. The total number of labeled cells was divided by the total number of glands seen in the image. The number of cells per gland was expressed as the mean ± SE. 

For estimating the labeling intensities of mucus (in pit and neck cells), TFF1 (in pit cells), TFF2 (in neck cells), pepsinogen (in chief cells) and H^+^,K^+^-ATPase (in parietal cells), images obtained from tissue sections probed with lectins or antibodies were loaded into the ImageJ densitometric software. In the case of immunoperoxidase labeling, image deconvolution was used to separate the DAB staining from the hematoxylin and/or periodic acid Schiff staining. Images were then converted to 8-bit and pixel density was calculated using the analysis tool. The percentage values obtained from the software were taken to reflect the amount of mucus/peptide/protein in the cells analyzed. Data were presented as mean ± SE. 

### 4.5. RT-qPCR Analysis

The total RNA was isolated from gastric mucosal tissues of control and VSL-treated mice using TRIzol reagent (Invitrogen) according to the manufacturer’s protocol. Then the cDNA was synthesized from 1µg of total RNA using High Capacity cDNA Reverse Transcription Kit (Applied Biosystems, Thermo Fisher Scientific, Baltics UAB, Vilnius, Lithuania) and Biometra Trio-Thermoblock^TM^. To study the differences in gene expression, SyBR green based (Applied Biosystems, Thermo Fisher Scientific, TX, USA) PCR was performed by keeping cDNA as template and primers specific for H^+^,K^+^-ATPase β-subunit, MUC-5ac, ghrelin, TFF1, TFF2, pepsinogen C (PgC), MUC6 and chromogranin A (CgA) were used as shown in Table 1. The QuantiStudio^®^ 7 Flex Real-Time PCR instrument (Applied Biosystems by Life Technologies) was used for amplification and quantification of dsDNA. Each sample was performed in triplicates and normalized with the housekeeping gene *GAPDH*. The Gene expression levels were calculated using the comparative cycle threshold method (∆∆C). Values were presented as mean ± SEM. *p* < 0.05 was considered significant.

### 4.6. Statistical Analysis

Results were presented as mean ± SE. Significance differences between control and VSL-treated groups were determined using the Student’s *t* test. A *p* < 0.05 was considered statistically significant.

## 5. Conclusions

Recently, the multi-strain probiotic VSL has been a subject of numerous clinical trials and studies that demonstrated its considerable potential for prevention or therapeutic applications in various gastrointestinal diseases. However, the mechanisms underlying the probiotic mode of action have not been fully elucidated. This research demonstrates for the effects of the probiotic mixture VSL on various cell lineages in the normal gastric mucosa of mice. Our results indicate that VSL enhances mucus, TFF1, and TFF2 production by both types of gastric mucous cells. While TFF2 was induced in chief cells, their production of pepsinogen was inhibited. These were associated with increased production of ghrelin secreting cells. Taken together, these data suggest the beneficial impact of VSL in the mouse stomach and its therapeutic value in diseases involving gastric epithelial cells.

## Figures and Tables

**Table 1:** List of primers used to quantify the expression of eight gastric cell lineage markers in VSL-treated versus control tissues.

Gene	Primer	Sequence (5′-3′)
GAPDH	Forward	TCAAGAAGGTGGTGAAGCAGG
	Reverse	TATTATGGGGGTCTGGGATGG
Muc5	Forward	AGG GCC CAG TGA GCA TCT CCTA
	Reverse	CAT CAT CGC AGC GCA GAG TCA
TFF2	Forward	GCA GTG CTT TGA TCT TGG ATG C
	Reverse	TCA GGT TGG AAA AGC AGC AGTT
HK-β	Forward	AAC AGA ATT GTC AAG TTC CTC
	Reverse	AGA CTG AAG GTG CCA TTG
Ghrl	Forward	AGGAATCCAAGAAGCCACCAGCTA
	Reverse	ATGCCAACATCGAAGGGAGCATTG
Muc6	Forward	CTC ACC TTC TAC CCC AGT ATC A
	Reverse	GGC AAC GAG TTA GAG TCA CAT T
TFF1	Forward	GGCCCAGGAAGAAACATGTATC
	Reverse	ACTGCTGGGCGGTGACA
PgC	Forward	AAACCGGCATCATGAAGTGGATGG
	Reverse	TTGTTCCTTCATGGTCTCCCGGAT
CgA	Forward	GCA GCA TCC AGT TCC CAC TTC C
	Reverse	TCC CCA TCT TCC TCC TGC TGA G

GAPDH: Glyceraldehyde 3-phosphate dehydrogenase; Muc5: Mucin 5ac; TFF2: trefoil factor 2; HK-β; HK-ATPase β; Ghrl: Ghrelin; Muc6: Mucin 6; PgC: Pepsinogen C; CgA: Chromogranin A.
